# Ptenb Mediates Gastrulation Cell Movements via Cdc42/AKT1 in Zebrafish

**DOI:** 10.1371/journal.pone.0018702

**Published:** 2011-04-11

**Authors:** Chen-Min Yeh, Yi-Ching Liu, Ching-Jen Chang, Shih-Lei Lai, Chung-Der Hsiao, Shyh-Jye Lee

**Affiliations:** 1 Institute of Zoology, National Taiwan University, Taipei, Taiwan, Republic of China; 2 Center for Biotechnology, National Taiwan University, Taipei, Taiwan, Republic of China; 3 Department of Life Science, National Taiwan University, Taipei, Taiwan, Republic of China; 4 Department of Bioscience Technology, Chung Yuan Christian University, Chung-Li, Taiwan, Republic of China; Baylor College of Medicine, United States of America

## Abstract

Phosphatidylinositol 3-kinase (PI3 kinase) mediates gastrulation cell migration in zebrafish via its regulation of PIP_2_/PIP_3_ balance. Although PI3 kinase counter enzyme PTEN has also been reported to be essential for gastrulation, its role in zebrafish gastrulation has been controversial due to the lack of gastrulation defects in *pten*-null mutants. To clarify this issue, we knocked down a *pten* isoform, *ptenb* by using anti-sense morpholino oligos (MOs) in zebrafish embryos and found that *ptenb* MOs inhibit convergent extension by affecting cell motility and protrusion during gastrulation. The *ptenb* MO-induced convergence defect could be rescued by a PI3-kinase inhibitor, LY294002 and by overexpressing dominant negative Cdc42. Overexpression of human constitutively active *akt1* showed similar convergent extension defects in zebrafish embryos. We also observed a clear enhancement of actin polymerization in *ptenb* morphants under cofocal microscopy and in actin polymerization assay. These results suggest that Ptenb by antagonizing PI3 kinase and its downstream Akt1 and Cdc42 to regulate actin polymerization that is critical for proper cell motility and migration control during gastrulation in zebrafish.

## Introduction

Gastrulation is a morphogenetic process involving cell migration and rearrangements to establish three germ layers: ectoderm, mesoderm, and endoderm [1]. In zebrafish, three distinct morphogenetic cell movements occur during gastrulation, including epiboly, involution, convergence and extension [2]. Gastrulation starts after the blastula stage when embryo proper appears as a mass of cells situated on top of yolk cells. The yolk sphere is then forming a dome cap that pushes the mass of blastomeres to become thinner and start to spread over the yolk sphere in a process called epiboly. After 50% of the yolk sphere is enclosed by the blastoderm, the front runner cells at the putative dorsal side begin to involute retrogradely toward the future anterior part to form mesoderm and endoderm progenitor cells. At about midgastrulation, convergence and extension movements occur to narrow medio-lateral and elongate anterior-posterior of body axis, respectively, that is essential to set up the dorsal-ventral and anterior-posterior axes [3], [4], [5]. These gastrulation cell movements are well demonstrated to be mediated by cell adhesion and cytoskeleton rearrangement [6], [7], [8].

Cell adhesion and cytoskeleton rearrangement can be associated to the metabolism of membrane lipids. One of the key enzymes for metabolizing membrane lipid is phosphoinositide 3-kinases (PI3 kinase). PI3 kinase can phosphorylate the D3 position hydroxyl group of the inositol ring of phosphatidylinositol-4,5-diphosphate (PIP_2_). Phosphorylation of PIP_2_ results in phophatidylinositol-3,4,5-triphosphate (PIP_3_) whose signaling is involved in cell proliferation, migration, survival, and apoptosis via Akt/PKB signaling [9]. Blocking PI3 kinase causes convergence and extension defects with reduced directional protrusions in leading cells of mesoderm in zebrafish [10]. This implies the possible involvement of PIP_2_/PIP_3_ balance in gastrulation cell movements. A counter enzyme of PI3 kinase is PTEN (Phosphatase and TENsin homolog deleted on chromosome 10, also named MMAC1 and TEP1), a famous tumor suppressor gene [11], [12]. Its mutations have been reported in numerous human cancers, like brain cancer, breast cancer, prostate cancer [13], melanoma [14], and some autosomal dominant cancer syndromes, like Cowden's disease [15], Bannayan-Zonana [16], and Lhermitte-Duclos disease [17]. PTEN has a phosphatase domain [18], which negatively regulates PI3 kinase/Akt pathway by dephosphorylating PIP_3_ [19], [20]. It regulates cell polarity during cell migration [21] by antagonizing PI3 kinase for a balance control of PIP_2_/PIP_3_ to mediate chemotaxis [22], [23]. It is thus intriguing to us to see how PTEN functions in concert with PI3 kinase during embryogenesis.

In mouse, fruit fly and chicken, PTEN is known to regulate cell migration, cell cycle length, and cell survival during early embryogenesis [24], [25], [26]. *Pten*−/− knockout mice die at around embryonic day 7.5 [24], [27] and altering *pten* expression before midblatula transition causes gastrulation delay in *Xenopus* embryos [28]. It appears that *Pten* is an indispensable gene during embryogenesis. However, the effects of *Pten* on the dynamic gastrulating cell movements have not been examined because of experimental constraints. Zebrafish is a well-established model to study the dynamic processes of gastrulation cell movements [29]. There are two zebrafish *pten* isoforms, *ptena* and *ptenb*. Each isoform has two alternatively splicing forms. Using the morpholino (MO) knockdown approach, Croushore et al. [30] showed that *ptena* or *ptenb* MO-injected embryos exhibit distinct morphological defects in 24–48 hours post fertilization (hpf), but the effects of those MOs on gastrulation were not described. Comparing genomic synteny, it reveals that zebrafish *ptenb* and the human *PTEN* have the conserved locus. It suggests that *ptenb* is probably the ortholog of human *PTEN* [31]. Thus, we set out to examine the effects of Ptenb on gastrulation cell movements and found that knockdown of *ptenb* by MO cell non-autonomously disturbs epiboly and convergent-extension cell movements during gastrulation in zebrafish.

## Results

### Ptenb expresses maternally and exists throughout early embryogenesis

**Figure 1:**
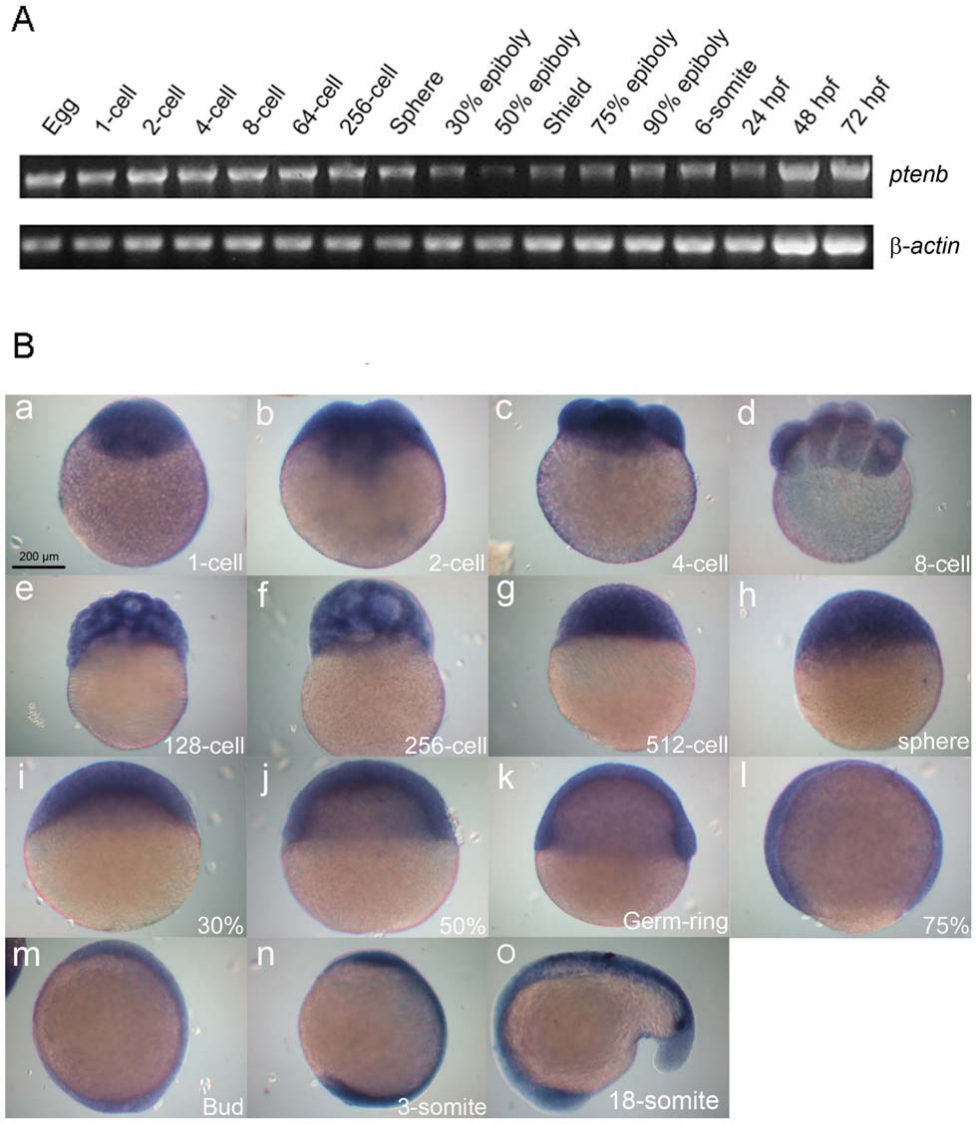
Expression patterns of zebrafish *ptenb*. (A) Expression of *ptenb* at designated developmental stage was examined by RT-PCR analysis of an 1183-bp *ptenb* fragment, and a 530-bp β-actin fragment was used as an internal control. hpf: h post fertilization. (B) Representative photographs of embryos fixed at designated stages and underwent whole-mount *in situ* hybridization against *ptenb*. Scale bar: 200 µm.

Previous studies have reported that *ptenb* is ubiquitously expressed in zebrafish early embryos at a few selected stages [30], [32], but its expression patterns at most cleavage and gastrulation stages are still lacking. RT-PCR analysis showed that *ptenb* mRNA was indeed expressed in every early embryonic stage examined, reduced at 30% epiboly then gradually recovered afterward (Fig. 1A). Whole-mount *in situ* hybridization (WISH) analysis revealed ubiquitous expression patterns of *ptenb* in embryos up to 18-somite stage (Fig. 1B). The maternal and ubiquitous expression patterns of *ptenb* during early embryonic stages suggested that it may play a pivotal role during early embryogenesis.

### Specificity and potency examination of translation-blocking *ptenb* MO

**Figure 2:**
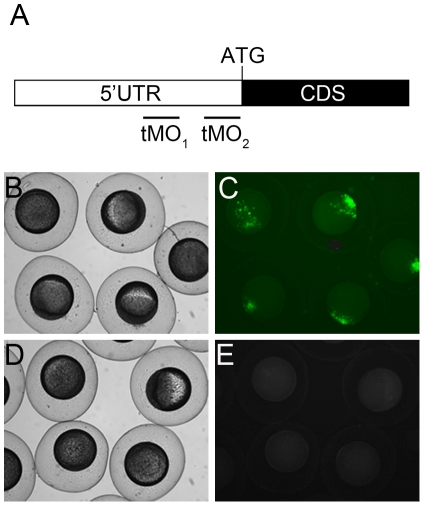
*ptenb* MO target sites and potency examination. (A) Partial mRNA map of *ptenb*. The black and white boxes represent part of the coding sequence (CDS) with ATG translation initiation site and 5′ untranslated region (UTR), respectively. The *ptenb* tMO_1_ and tMO_2_ binding sites are located at the 5′ UTR as shown. The potency of tMO_1_ to reduce *ptenb* expression was shown by co-injecting 150 pg PCS2+_*ptenb* 5′ UTR plasmid, which containing the tMO_1_ binding site, with or without 5 ng tMO_1_. Representative photographs of control (B,C) and morphant embryos (D,E) under bright (B,D) and dark fields (C,E) are shown. These experiments were repeated 3 times.

Two published antisense translational blocking MOs [30] were adopted to study the role of Ptenb during gasrulation. These two MOs bind to *ptenb* mRNA 5′ untranslated region (5′ UTR) at non-overlapping sites as indicated in Fig. 2A. To further confirm the specificity and translational blocking efficiency of the first *ptenb* MO (tMO_1_), we co-injected plasmids of a PCS2+ construct containing the tMO_1_ binding site (PCS2+_*ptenb* 5′ UTR) to check the tMO_1_ translation blocking ability. Embryos (n = 148) injected with the PCS2+_*ptenb* 5′ UTR plasmids alone expressed green fluorescent protein (GFP) in a mosaic pattern with a high ratio of 87.2±0.23% (N = 3) at 40% epiboly stage (Fig. 2C). By contrast, co-injection of 5 ng tMO_1_ (n = 160) completely blocked the expression of GFP (Fig. 2E). These results indicated that *ptenb* tMO_1_ specifically and potently blocks zebrafish *ptenb* translation.

### Knockdown of *ptenb* impairs gastrulation

**Figure 3:**
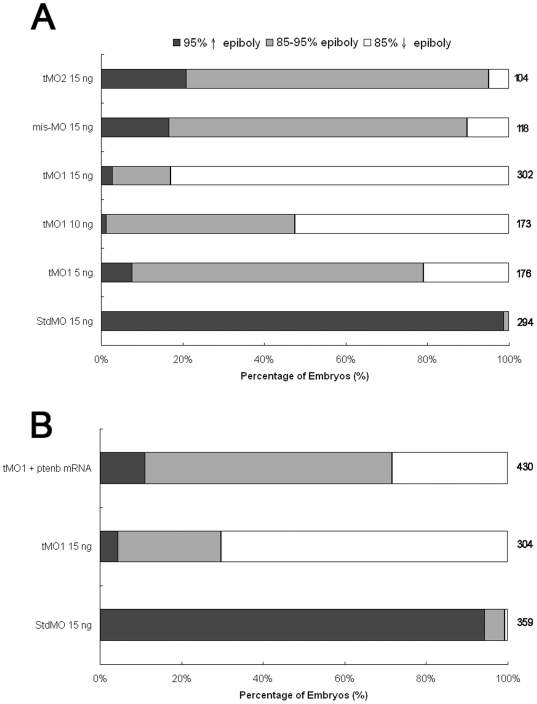
*ptenb* MOs delays epibolic progression. Knockdown of *ptenb* by MOs caused delay in epibolic movement in a dose-dependent manner. (A) Embryos were injected with standard MO (StdMO), tMO_1_, 5-bp mismatched tMO_1_ (mis-tMO1) and tMO_2_ at designated amount, incubated to 10 hpf and the percentages of embryos at each epiboly stage are shown. (B) Embryos were injected with 15 ng of StdMO or tMO_1_ in the absence or presence of *ptenb* mRNAs (50 pg), incubated and their degrees of epiboly progression are shown. Each treatment was repeated at least 6 times.

**Figure 4:**
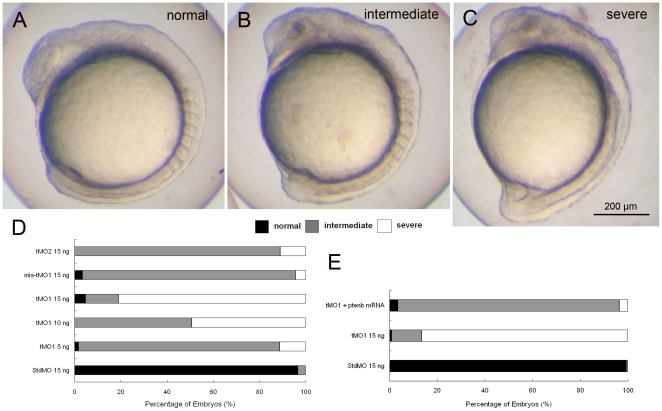
*ptenb* MOs specifically impairs gastrulation in a dose-dependent manner. Knockdown of *ptenb* by MOs caused gastrulation defects in graded severity. Representative photographs of *ptenb* morphants with different severity are shown in (A) normal (B) intermediate and (C) severe. (D) Embryos were injected with StdMO, tMO_1_, mis-tMO1 and tMO_2_ at designated amount, incubated to 10 somite stage and the percentages of embryos with different severity are shown. (E) Embryos were injected with 15 ng of StdMO or tMO_1_ in the absence or presence of *ptenb* mRNAs (50 pg), incubated and their degrees of gastrulation defect are shown. Each treatment was repeated at least 4 times.

To understand the role of Ptenb during early embryogenesis, we injected embryos with tMO_1_ and examined its effect on embryonic development. A standard control MO (StdMO), which has a minimum effect on embryonic development, was used as a MO control. The MO-injected embryos will be called morphants hereafter. The epibolic progression in *ptenb* morphants was notably slower and some morphants displayed a cylinder-like shape. Most of the StdMO morphants (98.7±0.9%) reached over 95% epiboly stage at 10 hpf (Fig. 3A). However, the epiboly progression was notably lagged in tMO_1_ morphants in a dose-dependent manner (N≧4). To test if the observed defects were specific to the loss of *ptenb*, we further tested the effects of a second published *ptenb* MO, tMO_2_ [30] and a 5-bp mismatched tMO_1_ (mis-tMO_1_). At a dosage of 15 ng, only 2.7±1.1% tMO_1_, 20.9±13.8% tMO_2_ and 16.6±11.7% 5-bp mis-tMO_1_ morphants reached over 95% epiboly stage at 10 hpf. Eventually (∼1–3 h lag) most of them could complete epiboly at MO dosages tested. This delay in epiboly-induced by *ptenb* MOs could be partially rescued by co-injecting *ptenb* mRNAs (Fig. 3B). We then examined the effect of *ptenb* MOs at 14 hpf and found the shortening of embryonic axis. The tail rudiment of tMO_1_ morphants was either less extended (intermediate phenotype, Fig. 4B) or stalled at vegetal pole (severe phenotype, Fig. 4C). This tail defect was tMO_1_-dose dependent (Fig. 4D). mis-tMO_1_ (15 ng)-injected embryos (4.5±1.6% severe and 92.0±1.9% intermediate phenotype, n = 201) exhibited a milder defect compared to tMO_1_ morphants (81.0±3.6% severe and 14.2±5.1 intermediate phenotype, n = 140). *ptenb* tMO_2_ (15 ng) also caused 11.0±1.6% severe and 89.0±1.6% intermediate defect (n = 168). Co-injection of 15 ng tMO_1_ with 50 pg *ptenb* mRNAs partially rescued the defects with 3.6±0.9% severe phenotype and 92.9±1.5% intermediate phenotype (n = 195, Fig. 4E). Taken together, the *ptenb* MO-induced defects appeared to be caused by the specific loss of *ptenb*. With the higher potency of tMO_1_ observed we used it at the rest of experiments unless otherwise stated.

**Figure 5:**
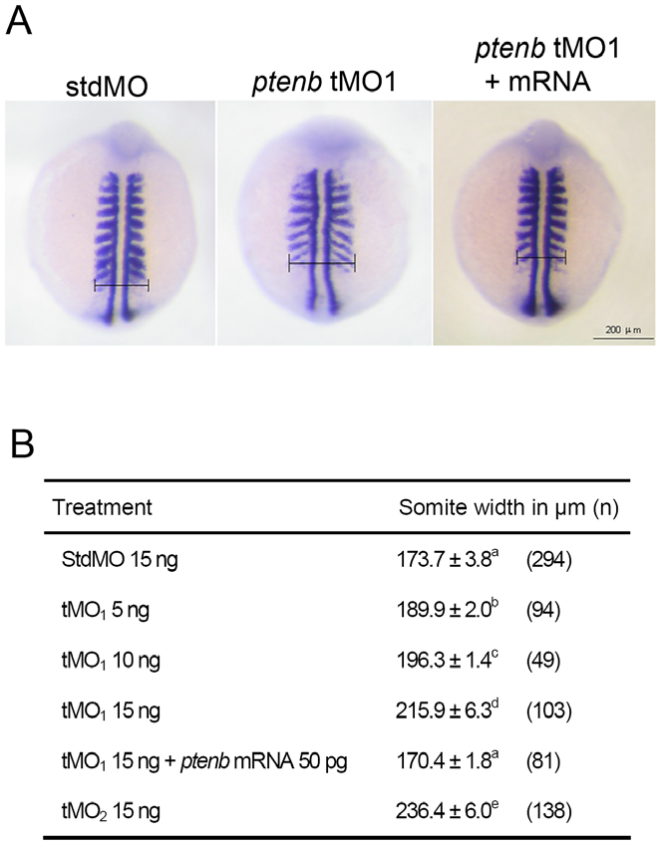
*ptenb* MOs cause convergence defect during gastrulation. Embryos were injected with designated amount of MO, incubated to 9 to 11 somites and subjected to WISH against *myod*. The somite width of treated embryos was measured as indicated in (A). The average somite width and number of embryos used (n) in each treatment are shown in (B). These experiments were repeated at least 4 times. Values between groups with a significant difference (p<0.05) are denoted by different superscript letters.

Since the observed shortening of embryonic axis is a typical convergent extension defect [33], we examined the effect of *ptenb* MO on convergence. Embryos were fixed after checking early morphological defects and subjected to WISH against *myod* to reveal somites. The width of the widest somite of the last three posterior somites was measured to estimate the extent of convergence (Fig. 5A). The average somite width was 173.7±3.8 µm in StdMO (15 ng) morphants, which was comparable to the untreated embryos (data not shown). Embryos injected with tMO_1_ showed expanded somite width in a dose-dependent manner (Fig. 5B). The somite width was 189.9±2.0 µm, 196.3±1.4 µm and 215.9±6.3 µm for embryos injected with 5 ng, 10 ng and 15 ng tMO_1_, respectively. tMO_2_ (15 ng) morphants also exhibited severe convergence defect with 236.4±6.0 µm in somite width. This convergence defect could be rescued by co-injecting 15 ng tMO_1_ and 50 pg *ptenb* mRNA with a somite width at 170.4±1.8 µm, which was similar to that of StdMO-injected embryos.

**Figure 6:**
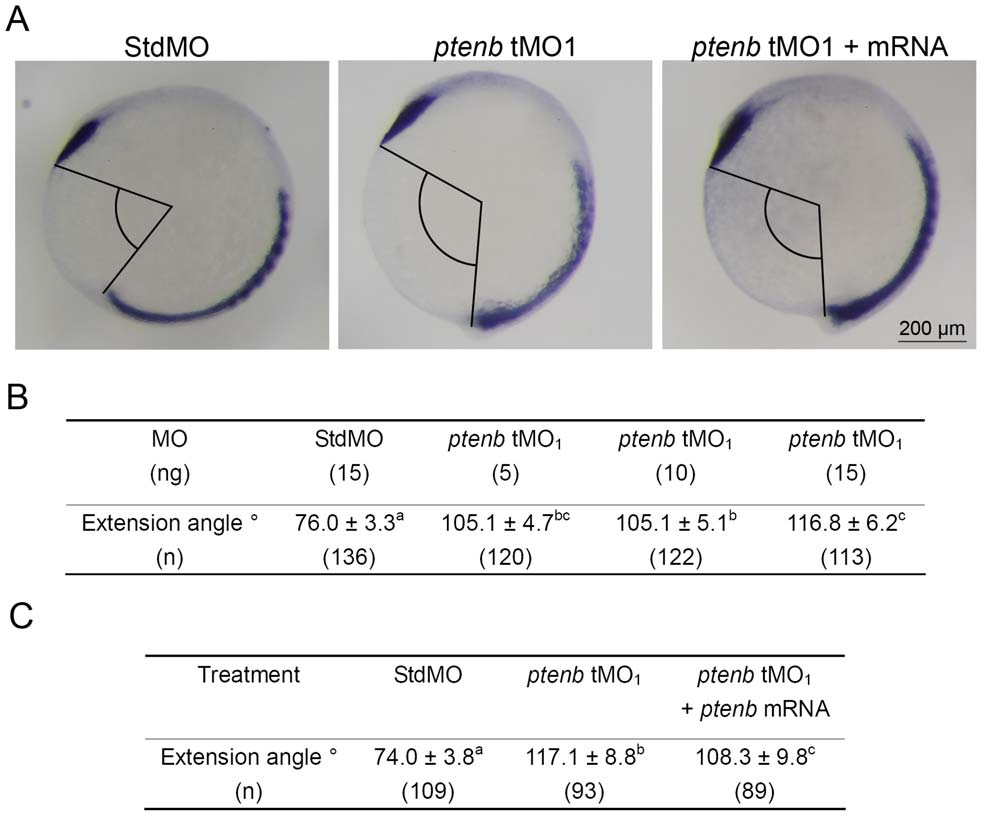
*ptenb* MOs cause extension defect during gastrulation. Embryos were treated as described in Fig. 5, but subjected to WISH against *ctsl1b* instead of *myod*. The extension angles of treated embryos were measured as indicated in (A). The average extension angle and number of embryos used (n) in each treatment are shown in (B). Rescue experiments were performed as shown in (C). These experiments were repeated at least 4 times. Values between groups with a significant difference (p<0.05) are denoted by different superscript letters.

To examine the effect of tMO_1_ on dorsal axis extension, we measured the angle between the anterior end of prechordal plate (as revealed by WISH against *ctsl1b*) and the posterior end of *myod*. The tMO_1_ morphants showed notable increase in angle (i.e. the inhibition of dorsal axis extension) compared to that of StdMO morphants (Fig. 6A). The *ptenb* MO-induced extension defect could also be partially rescued by *ptenb* mRNA co-injection as shown in Fig. 6B. Since the *ctsl1b* probe-labeled prechordal plates were observed in both control embryos and morphants (Fig. 6A), it appeared that the mesendodermal involution was not affected by the loss of Ptenb.

### Ptenb knockdown effects can be alleviated by inhibiting PI3 kinase

**Figure 7:**
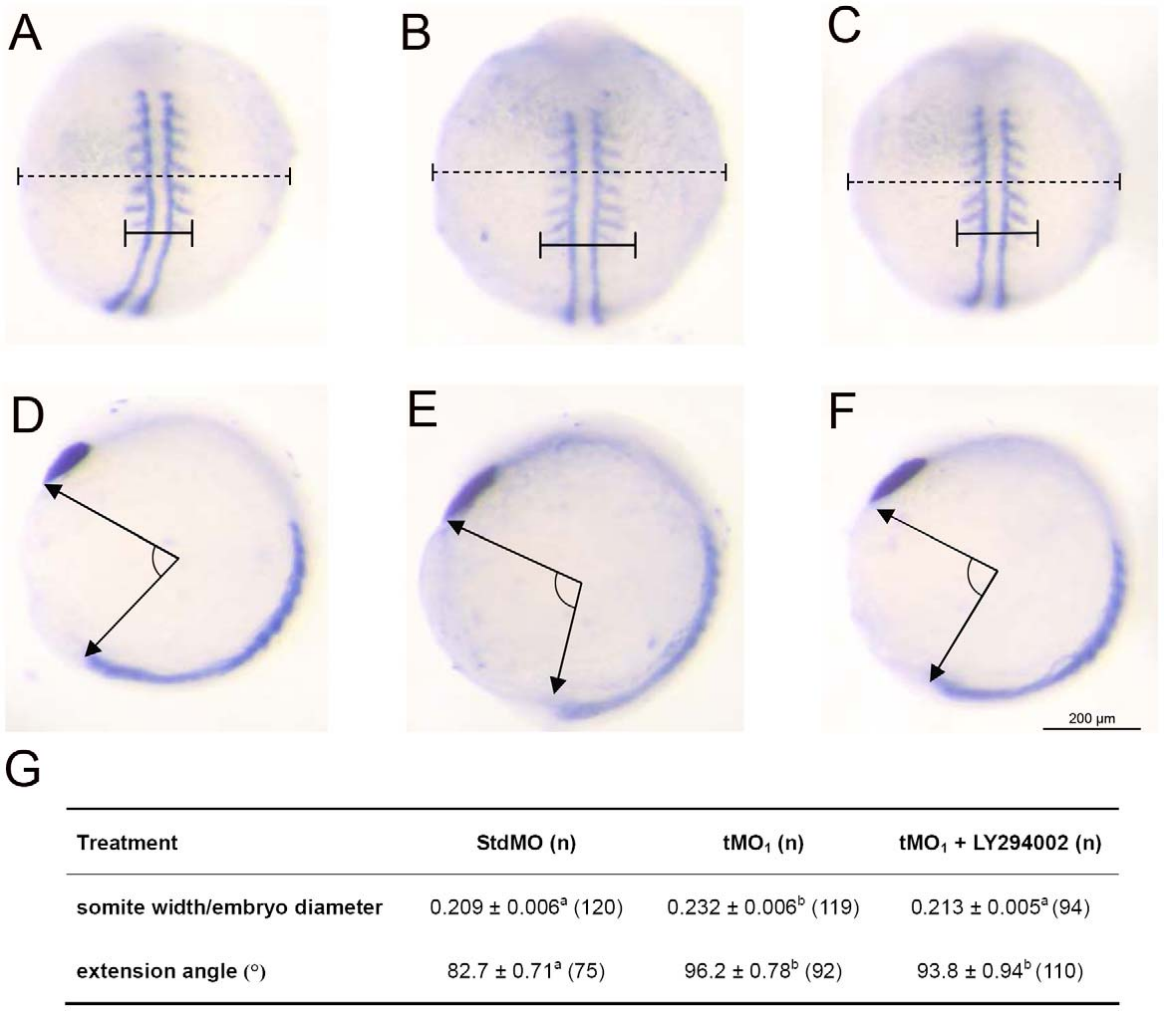
Rescue of *ptenb* MO-induced convergence defect by inhibiting PI3 kinase. Embryos were injected with 10 ng of StdMO (A,D) or tMO_1_ (B,E), incubated without (A,B,D,E) or with 5 µM of LY294002 (C,F) to 9 to 11 somites and subjected to WISH against *myod* and *ctsl1b*. The somite width (solid line), embryo diameter (dash line) (A–C) and extension angle (E–F) of treated embryos were measured as indicated. The average somite width/embryo diameter ratio, extension angle and sample number in each treatment are shown in (G). These experiments were repeated 5 times. Values between groups with a significant difference (p<0.05) are denoted by different superscript letters.

To understand whether *ptenb* MOs impair gastrulation cell movements through reinforcing PI3 kinase-mediated responses, we examined whether *ptenb* MO-induced gastrulation defects could be mitigated by inhibiting PI3 kinase. By analyzing the ratio of somite width to embryo diameter (Fig. 7A–C), we observed that in the presence of 5 µM of PI3 kinase inhibitor LY294002 (n = 72), the somite width ro embryo diameter ratio was not significantly affected (0.308±0.013 µm v.s. 0.295±0.003 µm, n = 85) in 10 ng StdMO-injected embryos (N = 3). By contrast, LY294002 almost fully rescued the impaired somite-convergent defect in tMO_1_ morphants (Fig. 7G). However, it appeared to be less potent to rescue the extension defect induced by tMO_1_ at the dosage tested (Fig. 7F,G). We attempted to use higher dosages up to 30 µm of LY294002, but the decorinated embryos were too fragile to be examined under those conditions. These results indicated that *ptenb* may regulate zebrafish convergent movement by antagonizing PI3 kinase.

### Ptenb regulates cell movements via modulating cell protrusive activity

**Figure 8:**
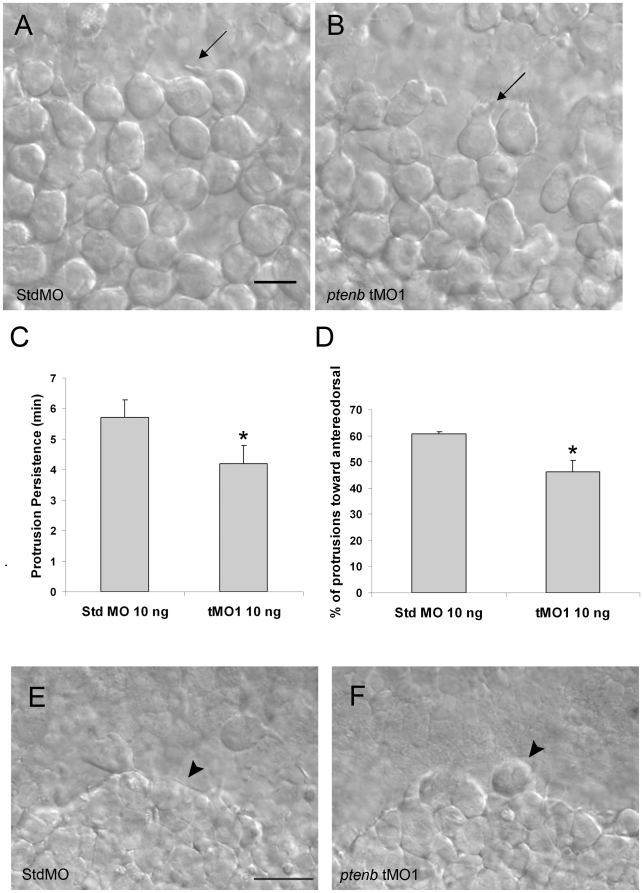
Cell protrusions of lateral hypoblast cells are affected by *ptenb* MO. Embryos were injected with 10 ng of StdMO (A) or *ptenb* tMO_1_ (B), imaged under DIC microscopy and recorded between 75% to 85% epiboly stages. Representative snapshots are shown and arrows are pointing to typical cell protrusions. By examining a 1-h recording, the average persistent time of each protrusion (C) and the percentage of protrusions pointed anterodorsally (D) were calculated and shown for each treatment. * : p<0.05, scale bars: 25 µm.

To examine how *ptenb* regulates convergence and extension cellular movements, we analyzed cell migration in different regions of gastrulating zebrafish embryos. As shown in movie S1 and S2, the leading edge hypoblast cells of a StdMO morphant formed different types of protrusion but mainly the long and thick lamellapodia at the lateral side where cells converge actively toward the dorsal side (arrow, Fig. 8A). In 4 trials, there was no significant difference in the number of lamellapodia formed per cell between StdMO (0.140±0.006/min, n = 28) and tMO_1_ (0.168±0.011/min, n = 29) morphants. By observing 29 lamellapodia at 75% epiboly stage in 4 StdMO morphants, we found these lamellapodia persisted for 5.8±0.6 min (Fig. 8C). More than half of these lamellapodia (60.7±1.0%, Fig. 8D) pointed toward anterodorsal direction. In contrast, although cells in tMO_1_ morphants also formed lamellapodia (n = 32) (Fig. 8B), the average persistence time was shorter (4.2±1.0 min, N = 4) compared with that in StdMO morphants (Fig. 8C) and only 46.2±5.0% (N = 4, Fig. 8D) of lamellapodia extended toward the anterodorsal direction.

The behaviors of prechordal plate leading edge cells at 90% epiboly were also analyzed for extension abnormality. In StdMO morphants, prechordal plate cells formed multiple layers and migrated as a sheet with the leading edge cells formed protrusions at front and adhered cells in the back. The leading edge cells (n = 17) were quite active, they formed protrusions, including filapodia, lamellapodia, and blebbing, and migrated fast over the yolk sphere at a velocity of 2.3±0.2 µm/min (N = 5, movie S3). The leading-edge cells of tMO_1_ morphants (n = 18) also had the same protrusions as those in StdMO morphants but their velocity of leading-edge cell migration was significantly slower at1.8±0.2 µm/min (N = 5, p<0.01, movie S4).

### Ptenb functions non cell-autonomously for gastrulation cell migration

**Figure 9:**
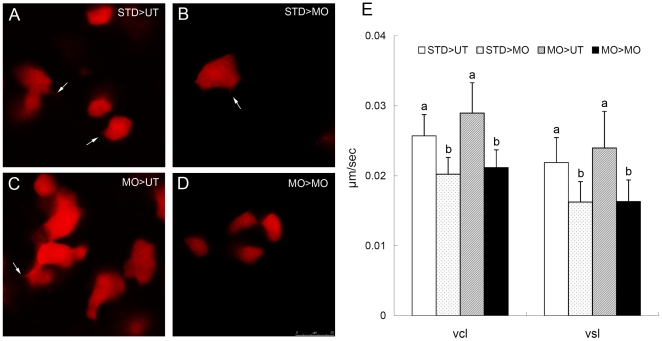
*ptenb* functions non cell-autonomously to regulate the cell protrusions and cell migration during gastrulation. (A–D) Rhodamine labeled blastomeres were transplanted from embryos injected with 10 ng of StdMO (STD) or tMO_1_ (MO) with rhodamine dextran to untreated hosts (UT) or tMO_1_ morphant hosts (MO). Host embryos were then imaged (animal pole on the top and vegetal pole at the bottom) under confocal microscopy and representative snapshots are shown in (A) STD>UT: StdMO treated cells in an untreated host. (B) STD>MO: StdMO treated cells in a tMO_1_ morphant. (C) MO>UT: tMO_1_ treated cells in an untreated host. (D) MO>MO: tMO_1_ treated cells in a tMO_1_ morphant. Arrows indicate the representative cellular protrusions in each embryo. (E) Cell transplantations were performed as described in (A–D), imaged under epifluorescent microscopy and time-lapse movies were taken for each treatment. The transplanted cells were traced and their curvillinear velocity (Vcl) and strait line velocity (Vcl) were calculated and shown. Values between groups with a significant difference (p<0.05) are denoted by different letters. Scale bar: 25 µm.

To assess cell-autonomy of *ptenb* function in mediating migration of lateral hypoblast cells, we performed cell transplantation experiments by using rhodamine dextran as a cell tracer. Blastomeres from StdMO or tMO_1_ morphants were transplanted to host embryos treated without or with tMO_1_, and examined under confocal microscopy or epifluorescent microscopy. Under confocal microscopy, cells from a StdMO morphant showed highly protrusive activities with well formed protrusions when transplanted into an untreated host embryo (“STD>UT”, arrow, Fig. 9A; movie S5) whereas protrusions were shorter and less well formed in those cells transplanted to a tMO_1_ morphant host (“STD>MO”, arrows, Fig. 9B; movie S5). Moreover, cells from a tMO_1_ morphant displayed a more polygonal shape (“MO>UT”, Fig. 9C; movie S5) when transplanted into an untreated host compared to those cells with a rounder shape and a reduced number of protrusions in a tMO_1_ morphant host (“MO>MO” Fig. 9D; movie S5). To examine the migration velocity and direction of transplanted cells in different hosts, we took time-lapse movies under epifluorescent microscopy for 6 embryos in each treatment and monitored about 8–10 cells in each embryo. The STD>UT cells traveled faster and longer than those STD>MO cells (Fig. 9E). The curvilinear velocity (Vcl, curvilinear distance/time) of STD>UT cells was 0.0257±0.0031 µm/sec (n = 61), but was only 0.0202±0.0024 µm/sec (n = 50), for the STD>MO cells (p<0.05). The MO>UT cells traveled with a faster speed and a longer distance in Vcl (0.0290±0.0043 µm/sec, n = 48) compared to those MO>MO cells (0.0212±0.0025 µm/sec, n = 54), but did not reach statistical difference (p>0.05). Furthermore, the STD>UT and MO>UT cells migrated straighter as shown by a higher straight line velocity (Vsl, straight line distance/time, Fig. 9E), which were 0.0219±0.0036 µm/sec (n = 61) and 0.0240±0.0053 µm/sec (n = 48), respectively. By contrast, and STD>MO (n = 50) and MO>MO (n = 54) cells migrated less linearly with a Vsl of 0.0163±0.0029 µm/sec (n = 50) and 0.0163±0.0031 µm/sec, respectively, compared to cells in the UT host. These results suggested that zebrafish Ptenb mediates gastrulation cell migration in a non cell-autonomous manner.

### Knockdown of *ptenb* enhanced actin polymerization

**Figure 10:**
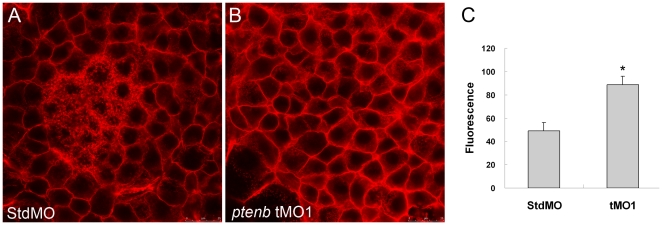
Knockdown of *ptenb* enhances actin polymerization. The 10 ng StdMO (A) and 10 ng *ptenb* tMO_1_ (B)-treated embryos were fixed at 70% epiboly, stained with rhodamine-phalloidin to reveal the F-actin, observed under confocal microscopy, and photographed with vegetal view. The F-actin contents of shield stage-embryos (n = 60) were measured by actin polymerization assay as described in “Materials and Methods”. Relative fluorescence (Rf) values from three independent experiments were corrected by subtracting background Rf. * indicates the value is significantly different from that of the StdMO treatment (p<0.05). Scale bar: 25 µm.

PTEN is known to control protrusion formation and adhesion during cell migration [34] via modulating actin dynamics [35]. To determine whether actin polymerization during gastrulation in tMO_1_ morphants was affected, we used rhodamine phalloidin staining to visualize filamentous actin (F-actin) in gastrulating embryos under confocal microscopy. The vegetal pole view showed that, F-actins were mainly located at cell boundaries corresponding to the cortical actin structure underneath cell membrane in StdMO-treated embryos (Fig. 10A). In contrast, the overall intensity of F-actin was enhanced without disturbing actin distribution patterns (Fig. 10B). To further confirm the increase of F-actin in tMO_1_ morphants, we measured the F-actin contents of these embryos by using an actin polymerization assay [36]. In 3 experiments, tMO_1_ morphants exhibited ∼1.8 folds of fluorescence compared to that of control embryos (Fig. 10C). These results demonstrated that knockdown of *ptenb* results in an increase in actin polymerization.

### Dominant-negative Cdc42 rescues *ptenb* MO-induced convergence and extension defects

Small GTPases Rac1 and Cdc42 were known downstream of PTEN in *in vitro* studies [35]. To examine whether Cdc42 and/or Rac1 are involved in the Ptenb-mediated regulation in gastrulation cell movements, dominant negative, T17NCdc42 or T17NRac1 were used to see their effects on the *ptenb* MO-induced convergent extension defects. Co-injection of T17NCdc42 mRNAs (25 pg) with StdMO (10 ng) did not change the somite width, but increased the extension angle (N = 4, Fig. S1A). In contrast, T17NCdc42 mRNAs could partially restore the somite width and extension angle of tMO_1_ morphants (Table 1, N = 4). Similar experiments were performed using T17NRac1 (Table 1, N = 3). Co-injection of T17NRac1 mRNAs (50 pg) with StdMO (10 ng) caused 93.0%±0.0% embryos with severe epiboly arrest (Fig. S1C,D) compared to 2.4%±0.0% in embryos-injected with StdMO only (Fig. S1B,D, N = 4). T17NRac1 mRNAs (50 pg) and *ptenb* tMO_1_ (10 ng) co-injected embryos only slightly improved the convergent extension defect compared to that of tMO_1_ morphants with no statistical difference (p>0.05) between groups.

**Table 1:** The *ptenb* MO-induced convergent extension defects can be rescued by dominant-negative Cdc42.

	Somite width (µm)(n)	Extension angle (°)(n)
StdMO 10 ng	204.9±1.7^a^(155)	71.6±1.8^a^(157)
*ptenb* tMO_1_ 10 ng	228.9±4.5^b^(105)	113.9±8.7^b^(105)
*ptenb* tMO_1_ 10 ng+T17NCdc42 25 pg	212.6±2.6^c^(85)	98.9±3.6^c^(87)
StdMO 10 ng	192.0±4.6^a^(124)	71.2±4.0^a^(124)
StdMO 10 ng+T17NCdc42 25 pg	192.6±4.0^a^(92)	79.3±4.6^b^(92)
StdMO 10 ng	172.9±4.5^a^(75)	75.5±8.3^a^(75)
*ptenb* tMO_1_ 10 ng	195.6±4.4^b^(91)	115.0±1.9^b^(92)
*ptenb* tMO_1_ 10 ng+T17NRac1 50 pg	188.7±5.0^b^(112)	110.5±4.5^b^(114)

Embryos were injected with StdMO and *ptenb* tMO_1_ in the presence or absence of T17NCdc42 or T17NRac1 mRNAs, subjected to WISH against *myod* and *ctsl1b* and somite width and extension angle of embryos were measured (N≧4).

### Overexpression of human constitutively active AKT1 causes convergence and extension defects

Knockdown of *ptenb* would lead to increase of downstream Akt/PKB activity, to investigate whether the elevation of AKT/PKB activity may result in similar *ptenb* MO-induced phenotypes. We overexpressed human constitutively active *akt1* (*caakt1*) in zebrafish embryos to observe its effect on convergent extension. Embryos-injected with 1× buffer and *caakt1* mRNAs at 50, 100, or 200 pg were collected and subjected to WISH against *myod* and *ctsl1b*. As shown in Table 2, lower doses (50 and 100 pg) of human *caakt1* mRNAs caused slight convergent extension defects, but profound inhibition on convergent extension was observed in embryos-treated with 200 pg *caakt1* mRNAs. These results implied that the *ptenb* MO-induced convergent extension defects might be due to the elevation of AKT1 activity.

**Table 2:** Constructively-active human AKT1 cause convergent extension defects in zebrafish embryos.

	Somite width (µm)(n)	Extension angle (°)(n)
1× buffer	173.7±3.3^a^(78)	77.7±5.3^a^(79)
*caakt1* 50 pg	176.4±2.0^b^(96)	84.0±4.0^b^(97)
*caakt1* 100 pg	175.9±2.7^b^(85)	82.0±5.8^b^(89)
*caakt1* 150 pg	202.4±3.0^c^(75)	113.0±3.7^c^(82)

Embryos were injected without or with different amount of constructively-active human *akt1* (*caakt1*) mRNAs, subjected to WISH against *myod* and *ctsl1b* and somite width and extension angle of embryos were measured (N = 4).

### Ptenb MO causes convergence and extension defects in *ptenb*−/− embryos

The lack of notable early embryonic defect in *ptenb*−/− mutant fish is in contrast to our observations described above that might be due to the presence of maternal *pten* transcripts [32]. To examine this possibility, we had obtained heterozygous *ptenb*+/− embryos, raised to adults to give rise to F_1_ offspring. Those F_1_ fish were screened by PCR for the presence of mutated *ptenb* to get *ptenb*−/− homozygous fish according to Faucherre et al. [32]. Different dosages of *ptenb* tMO_1_ were injected into *ptenb*−/− embryos, fixed and subjected to WISH against *myod* and *ctsl1b*. Similar results of *ptenb* MO-induced convergent extension defects were obtained as that of wild type embryos (Table 3). Extension angle and somite width of *ptenb*−/− embryos were significantly increased in the presence of *ptenb* tMO_1_. These data implied that the presence of maternal *ptenb* transcripts was sufficient to complete normal gastrulation in *ptenb*−/− embryos.

**Table 3:** The *ptenb* MO induces convergent extension defects in ptenb homozygous mutant.

Treatment	Somite width (µm)(n)	Extension angle (°)(n)
StdMO 10 ng	222.4±6.5^a^(137)	65.6±2.5^a^(144)
*ptenb* tMO_1_ 5 ng	231.2±6.3^b^(n = 126)	92.8±4.2^b^(126)
*ptenb* tMO_1_ 10 ng	238.0±5.9^c^(138)	95.4±6.2^b^(151)

Embryos collected from homozygous *ptenb* mutants were injected with StdMO or *ptenb* tMO_1_, subjected to WISH against *myod* and *ctsl1b* and somite width and extension angle of embryos were measured (N = 4).

## Discussion

The necessity of PTEN in embryogenesis has been demonstrated in several animal species including mice, chickens and fruit flies [24], [25], [26]. However, the role of Pten in zebrafish gastulation has been controversial. In this study, we demonstrated that *ptenb* may regulate zebrafish gastrulation cell movements by controlling actin polymerization and directed cell migration via antagonizing PI3 kinase and downstream Cdc42 and Akt activity.

### Ptenb regulates convergence and extension during gastrulation

PI3 kinase has been shown to be required for cell polarization of gastrulating mesendodermal cells by overexpressing dominant-negative form of PI3 kinase or using the PI3 kinase inhibitor LY294002 [10]. The authors unequivocally demonstrated the necessity of maintaining PIP_2_/PIP_3_ balance in mesendodermal cell polarity of directed migration during gastrulation in zebrafish. It is logical to hypothesize that the negative regulator of PI3 kinase, PTEN, should also be necessary for regulating gastrulation cell movements in zebrafish, but no gastrulation defects was observed in zebrafish *pten* mutants [37]. Based on the *pten* mutant study, Pten appears to play no role during gastrulation in zebrafish. However, the presence of maternal *pten* messages in early mutant embryos cannot be excluded. Secondly, the lack of functional Pten in the zebrafish *pten* mutants have not be demonstrated due to the lack of antibodies which can recognize zebrafish Pten. Therefore, we took an alternative approach by using *pten* MOs which have been previously shown to have specifically lowered *pten* translation and enhanced AKT activity [30] to analyze their effects on gastrulation cell movements in zebrafish. Our results clearly indicated that block of *ptenb* activity affects convergent extension movements during gastrulation in zebrafish.

### Zebrafish Ptenb regulates gastrulation in coordination with PI3 kinase

Pten is a known counter enzyme of PI3 kinase by dephosphorylating PIP_3_ to PIP_2_ [9], [18], and also acts via downstream effectors AKT/PKB to regulate early developmental processes [22], [25], [28]. To examine the coordination of PI3 kinase and Ptenb in regulating zebrafish gastrulation, we showed that LY294002 could rescue convergence defect in tMO_1_ morphants. It clearly demonstrated that PI3 kinase and PTEN linkage is required to modulate cell movements during zebrafish gastrulation.

### Zebrafish Ptenb regulates protrusive activities of lateral mesendodermal cells but not prechordal plate cells during gastrulation

In the LY294002 rescue experiment, the result suggested that *Ptenb* coordinates with PI3 kinase to regulate gastrulation cell movements presumably via their regulation on PIP_2_/PIP_3_ balance. Activated PI3 kinase can increase PIP_3_ concentration, which would localize at the frontal edge of protrusions and induce the directional migration, and Pten acts at the rear edge of cells to dephophorylate PIP_3_ to PIP_2_ then restrict the spatial expression of PIP_3_ in the cell [22], [23]. Our time-lapse recordings clearly demonstrated that active protrusive activity is required for cell movements during zebrafish gastrulation. The *ptenb* MO-induced gastrulation defect was not due to the change in protrusion numbers. In stead, the directionality and persistency of protrusion were more relevant. Lamellapodia were formed at the leading edge of lateral hypoblast cells and these protrusions were mainly pointing anterodorsally to the direction of cell movement. However, tMO_1_ morphants' lateral hypoblasts exhibited interference in persistence and directionality of protrusions. In the lateral hypoblasts of tMO_1_ morphants, the increase of lamellapodia turnover was evident by the reduction of lamellapodia persistence. At the same time, the directionality was also affected by decreasing the number of protrusions pointed anteriodorsally. The directional defect was also observed at the transplantation experiment. StdMO cells transplanted to untreated embryos revealed a better linearity than the tMO_1_ morphant cells transplantated to tMO_1_ hosts. The abnormality of protrusion persistence and migration directionality was not observed at prechordal plate cells, this result might be due to the fact that prechordal plate cells migrate as multicellular sheets and cell-cell are highly contact. The rear area of the leading edge cells were in close contact with other cells that might help to maintain cell polarity in the absence of Ptenb [38].

### Zebrafish Ptenb mediates gastrulation cellular movements via controlling actin polymerization and downstream Akt1 and Cdc42

*In vitro* cell studies have shown that, the increase of PIP_3_ concentration in cells activates Rac1 and Cdc42 which would lead to actin polymerization and lamellapodia and filapodia formation [35], [39]. Rac1 has further reported to be important in *Drosophila* mesoderm migration during gastrula [40] and zebrafish convergence by controlling lamellapodia formation [41]. In our results, dominant negative Cdc42 and to a less extent of dominant negative Rac1 also showed to rescue convergent extension defects in *ptenb* morphants.

AKT/PKB is another known downstream factor of PI3 kinase. The binding of AKT/PKB to PIP_3_ can recruit it to membrane for the regulation of actin arrangement and protrusion formation [42]. Knockdown of *ptenb* should lead to an increase of PIP_3_ and subsequent elevation of AKT activity in zebrafish embryos. The elevated AKT activity might disturb the actin cytoskeleton, protrusion formation and cell movements. Over-expression of *caakt1* showed convergence and extension defects. These results demonstrated that Ptenb regulates zebrafish convergence and extension during gastrulation via PI3 kinase-Akt pathway that is consistent with the rescue of *ptenb* tMO_1_ morphants by the PI3 kinase inhibitor.

Gastrulation cell movements are tightly regulated by actin cytoskeletons. Here, we showed that actin polymerization was enhanced about two folds in the *ptenb* tMO_1_ morphants than the control embryos. These results suggest that zebrafish Ptenb downregulates small GTPases Rac1 and Cdc42, which further rearrange actin polymerization, then controls the convergence and extension during gastrulation.

### *ptenb* MO induces convergence and extension defects in *ptenb*−/− embryos

The lack of gastrulation defect in the *pten* mutants argues a role of PTEN in zebrafish and the authors also questioned that the effects of *pten* MOs on zebrafish early embryos reported by Croushore et al. [30] may not be due to the specific loss of Pten [32]. However, we demonstrated the specificity of *ptenb* MO used with the following evidences: (1) Co-injection of *ptenb* tMO1 and ptenb 5′ UTR-GFP plasmid showed no fluorescence signal compared to ptenb 5′ UTR-GFP injection alone. (2) *Ptenb* tMO_1_ caused convergence and extension in a dose-dependent manner. (3) The defects of *ptenb* morphants could be rescued by exogenous *ptenb* mRNAs, LY294002 and dominant-negative Cdc42. (4) Since the generation of functional truncated Ptenb protein could not be excluded in the *ptenb* mutants [32], the *ptenb*−/− embryos was treated with *ptenb* tMO_1_ and these mutant embryos showed similar morphological defects with wild type embryos. In addition to our results, Croushore et al., [30] had shown that *ptenb* tMO_1_ inhibited Ptenb protein *in vitro* translation assay and elevated phospho-Akt (pAkt). Those results supported that the *ptenb*-induced gastrulation defects observed were specifically due to the loss of *ptenb*.

**Figure 11:**
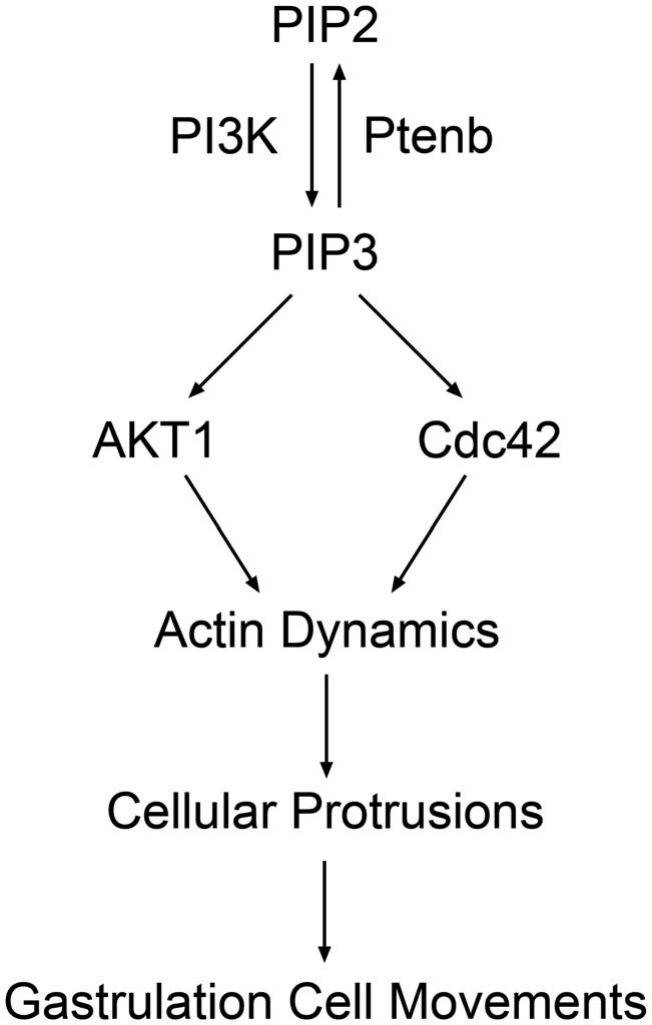
Ptenb-mediated signal transduction during gastrulation in zebrafish. By coordinating with PI3 kinase, Ptenb controls the balance between PIP_2_ and PIP_3_ that may activate downstream Cdc42 and AKT1 for mediating actin dynamics to regulate cell movements during gastrulation in zebrafish.

In summary, we provide the first *in vivo* evidence that *Ptenb* coordinates with PI3 kinase to modulate downstream AKT1 and Cdc42 for rearranging actin polymerization and protrusion formation that can lead to proper cell migration to regulate convergence and extension cell movements during gastrulation in zebrafish as schematically depicted in Fig. 11.

## Materials and Methods

### Ethics Statement

All animal handling procedures were approved by the use of laboratory animal committee at National Taiwan University, Taipei, Taiwan (IACUC Approval ID: 97 Animal Use document No. 55).

### Zebrafish maintenance and embryo collection

Wild-type TU/AB zebrafish were inbreeded and maintained in a 14-hr light/10-hr dark cycle and 28.5°C incubator. Embryos were collected by natural spawning and raised in 0.3× Danieau's buffer (diluting by 1× Danieau's buffer: 58 mM NaCl, 0.7 mM KCl, 0.4 mM MgSO_4_, 0.6 mM Ca(NO_3_)_2_, and 5.0 mM HEPES (pH 7.6), with double distilled water) until observation or fixation. The definition of embryo stage was according to Kimmel et al. [43], and the stages are indicated as hours post-fertilization (hpf).

### RNA isolation and RT-PCR analysis

Zebrafish RNAs of selected embryonic stages were isolated by TriSolution Reagent Plus (GeneMark, Atlanta, Georgia, USA). RT-PCR was applied for cDNA synthesis according to the manufacturer's instructions. cDNAs were generated from total RNAs and served as templates for an 1183-bp fragment of *Ptenb* amplification with the following primers: 5′-GGCTGCGATCATAAAGGAAT-3′ (forward) and 5′-CTGTTCTTCATCGTACTGTTCA-3′ (reverse). A 530-bp β-actin amplified with primers: 5′-TTGGTATGGGACAGAAAGACAGCTAC-3′ (forward) and 5′-AAGGGCCACATAGCAGAGCTTC-3′ (reverse) was used as a RT-PCR control.

### Morpholino oligonucleotide microinjections

All antisense morpholino oligonucleotides (MOs) were custom made by Gene Tools, LLC (Philomath, OR). Standard control MO (Std MO, 5′-CCTCTTACCTCAGTTACAATTTATA-3′) has no homology sequence to any known zebrafish sequence which was used. Two published *ptenb* translational blocking MOs, tMO_1_ and tMO_2_ were used with the following sequences: *Ptenb* tMO_1_ (5′-CTTTCGGACGGTCGGTCGTCTTTA-3′), which targeting from −74 to −51 of the 5′ UTR region; *Ptenb* tMO_2_ (5′- GGCTGTGACAGGAG-TCTTTAGGGTT-3′), which targeting from −25 to −1 [30]. A 5-base mismatch tMO_1_ was designed (5′ -CTTTgGGACcGTCcGTgGTgTTTA-3′), the mismatched bases were indicated by subscripts. All MOs were dissolved in sterile mini Q water to 1 mM stocks and stored at room temperature. MOs were diluted to proper working concentration by 1× Danieau's buffer with 0.5% phenol red and keep in room temperature. Thin-wall 3.5 inch glass capillaries (1.14×0.50 mm, O.D.×I.D., World Precision Instrument, Sarasota, FL) were pulled using a horizontal puller (P-97, Sutter Instrument, Navato, CA). Embryos at one-cell stage were stationary at an injection tough on a 100-mm 1% agar plate. MOs were diluted as described at desired concentrations and loaded into a pulled capillary. The tip of loaded capillary was forced through the chorion and into the yolk cells to reach the junction between yolk cells and blastodisc where the MO was ejected by using an oil Nanoliter injector (Nanoliter 2000, World Precision Instruments) at 2.3 nl. After injection, embryos were take out from the injection troughs and cultured in 0.3× Danieau's buffer at 28.5°C until being examined.

### Cloning and preparing mRNA

After RNA isolation, *ptenb* cDNA was obtained by RT-PCR using primers: 5′-CCGCTCGAGATGGCTGCGATCATAAAGGAATTTG-3′ (forward) and 5′-GAATTCTCAAACTTTAGTAATCTGTTC-3′ (reverse), then cloned into pGEM-T easy vector. The clone was further subcloned into an expression vector, pcGlobin2 [44], by NotΙ digestion. The *ptenb* construct was linearized by XbaΙ, and the capped RNA was transcribed according to the manufacturer's instruction using a mMESSAGE mMACHINE® T7 Kit (Applied Biosystems, Carlsbad, CA) according to manufacturer's instructions. The dominant-negative zebrafish Rho-GTPase, T17NCdc42 and T17NRac1, were cloned separately into pGEM-T easy vector (Promega, Madison, WI) by using a QuikChange Site-Directed Mutagenesis kit (Agilent Technologies, Carlsbed, CA). The primers for wild type zebrafish Cdc42 are: 5′-ATGCAGACGATCAAGTGCGT-3′ (forward), 5′ ACGCTCATAGCAGCACACAT-3′ (reverse) ; and for wild type Rac1 are: 5-ATGCAGGCCATAAAGTGTGT-3′ (forward), 5′-CAGAAGGAGACATCTTCTCC-3′ (reverse); after cloned the wild type Cdc42 and Rac into pGEM-T easy, the primers with mutation sites were used to obtain the dominant negative zebrafish Cdc42 (T17NCdc42) and Rac1 (T17NRac1) constructs by performing PCR again. The primers with mutation sites of Cdc42 are: 5′-GATGGTGCAGTGGGTAAAAACTGTCTA-3′ (forward); 5′-GTATAGGAGATTAATAGACAGTTTTTA-3′ (reverse). The primers with mutation sites of Rac are: 5′-GGGCTGTGGGAAAAAACTGCCTTCTGATCAGC-3′ (forward); 5′-GCTGATCAGAAGGCAGTTTTTTCCCACAGCCC-3′ (reverse). The T17NCdc42 and T17NRac1 were linearized by SalΙ and ApaΙ then transcribed into mRNA by mMESSAGE mMACHINE® Sp6 and T7 Kits, respectively. The human constitutively active human Akt1 (*caakt1*) was constructed by Gataway cloning to make pCSDEST-myrAkt1 from a myrAkt delta4–129 plasmid (Addgene, Cambridge, MA). The *caakt1* plasmids were digested by SacΠ and mRNAs were synthesized by Sp6 Kit.

### Whole-mount *in situ* hybridization (WISH)

Embryos were fixed in desired stages in 4% paraformaldehyde in phosphate-buffered saline overnight, dechorionated manually by fine forceps and stored in 100% methanol at −20°C until use. Antisense digoxigenin (DIG)-labeled RNA riboprobes were synthesized following the manufacture instructions (Roche Applied Science, Penzberg,Germany). The *myod* construct [45] was linearized by XbaΙ and transcribed by T7 RNA polymerase (Roche Applied Science, Penzberg, Germany). The *ctsl1b* construct [46] was linearized by NotΙ and transcribed by T7 RNA polymerase. *In situ* hybridization and detection were performed according to Thisse et al. [47] with a phosphatase-coupled anti-DIG antibody. The processed embryos were then transferred to 100% glycerol and photographed by using Nikon CoolPIX 995 digital camera.

### Measurement and counting of embryos

For the convergence and extension assay, *ptenb* MOs or STD MO treated embryos were incubated at 28.5°C until 8 to 11-somite stages for morphological observation under stereoscope. After the morphology classification, the embryos were fixed and subjected to WISH against *myod* to measure the width of *myod* signal of the widest somite among the last three somites. Furthermore, the extension defects were characterized by measuring the angle between prechordal plate staining using *ctsl1b* and the vegetal end of *myod* signals. All experiments were repeated at least three times and the measurements were made using tools in Adobe Photoshop CS4

### DIC time-lapse cell migration recording

To monitor the migration of lateral hypoblast leading edge cells (marginal deep cells), the embryos at 75% epiboly stage were dechorionated with 0.01 mg/mL protease (Sigma, St. Louis, MO) and mounted in 0.8% low-melting agarose (Amresco, Solon, OH). The migrating cells were recorded using a 40× water immersion objective under a Leica DM5000 B DIC microscope (Leica Microsystems, Wetzlar, Germany). One hour long movies were recorded at 30-sec intervals by a CoolSNAP fx CCD camera (Roper Scientific, Tucson, AZ). The movies were acquired and analyzed by Simple PCI Imagine System software (Compix, Sewickley, PA). The protrusions which merged from cell body with an angle <135°C and a width >2 µm were defined as a lamellapodium extension. To monitor the migration of prechordal plate cells, the embryos at 90% epiboly stage were dechorionated and mounted as described, and then monitored, filmed (5 min movies at 5 sec intervals) and analyzed using a 63× water immersion as described. To track transplanted cells, 1 hour long time-lapse recordings with 10-sec intervals were taken and analyzed by using the Simple PCI software for migration path, velocity and linearity (the shortest distance between the start and end points of the movement divided by the total distance moved) analyses.

### PI3-kinase inhibitor (LY294002) treatment

Embryos treated with *Ptenb* tMO_1_ were dechorionated at 30% epiboly stage and treated with 5 µM LY294002 (Calbiochem, Darmstadt, Germany) from 30% epiboly to 90% epiboly in E3 media and transferred to 0.3× Danieau's buffer until 7 somite stage and collected for further *in situ* hybridization examination.

### Actin polymerization assay

The filamentous actin (F-actin) of embryo was determined by fluorometric assay which based on the binding affinity of rhodamine phalloidin and F actin [48], [49]. Embryos injected with different MOs were fixed at shield stage in 5% formaldehyde in Actin Stabilizing Buffer (ASB) [50] overnight. The embryos were then incubated in 150 mM glycine-ASB for 3 hours, washed by ASB, and then labeled by 165 nM rhodamine phalloidin (Molecular Probes, Inc., Eugene, OR). Control embryos were incubated by 20 mg/mL unlabeled phalloidin for 50 minutes prior to rhodamine phalloidin staining. All samples in a group of 60 embryos were then washed in ASB and extracted in dark with methanol and homogenized by 24G needles, and incubated with rotating for 36 hours at 4°C. The fluorescence signals were detected from the extractant and measured by using a fluorospectrophotometer (Hitachi F-2000, Tokyo, Japan) with an excitation wavelength of 565 nm and an emission wavelength of 580 nm [36].

### Cell transplantation

For transplantation preparation, donor embryos were injected with 0.5% rhodamine dextran and Std MO 10 ng or *Ptenb* tMO_1_ 10 ng respectively. Donor embryos were transplanted into wild type and *Ptenb* morphant host separately at sphere stage as described previously [51]. Transplanted cells were further recorded by using Leica DM5000 B microscope and Leica TCS SP5 Confocal Microscope Imaging System.

### *ptenb*−/− zebrafish screening

Heterozygous *ptenb*+/− zebrafish embryos were kindly provided by Jeroen den Hertog (Hubrecht Institute, Utrecht, The Netherlands). Those embryos were raised to adults and mated to produce F_1_ fish. Nested PCR was performed to select homozygous *ptenb* −/− zebrafish according to Faucherre et al. [32].

### Statistical analysis

All experimental values are presented as mean ± standard error and were analyzed by unpaired-sample Student's t-test in Microsoft Excel. N indicates the number of experiments repeated; n indicates the total sample number in one experimental condition. Different superscript lettering between values stands for a significant difference at p<0.05.

## Supporting Information

Figure S1 **Embryos were injected with 10 ng of StdMO with or without 25 pg of T7 NCdc42 mRNAs and treated as described in** **Table 1** **.** The somite width and extension angle of each embryo were measured and shown (A). Embryos were injected with 10 ng of StdMO without (B) or with 50 pg of T7 NRac1 mRNAs (C), incubated to 10-somite stage and photographed. The percentages of normal and abnormal embryos in each treatment are shown (D).
(PDF)

Movie S1 **Time-lapse imaging of cellular migration and protrusions in lateral hypoblast cells of a StdMO-injected morphant.** Shown here is a 1-h at 30-s intervals DIC time-lapse image sequence of a 75% epiboly stage embryo-injected with 10 ng StdMO. The animal pole is on the top and the dorsal side is to the left.
(AVI)

Movie S2 **Time-lapse imaging of cellular migration and protrusions in lateral hypoblast cells of a** ***ptenb*** **tMO_1_-injected morphant.** Shown here is a 1-h at 30-s intervals DIC time-lapse image sequence of a 75% epiboly stage embryo-injected with 10 ng *ptenb* tMO_1_. The animal pole is on the top and the dorsal side is to the left.
(AVI)

Movie S3 **Time-lapse imaging of cellular migration and protrusions in involuting prechordal plate cells of a StdMO-injected morphant.** Shown here is a 5-min movie at 5-s intervals DIC time-lapse image sequence of a 90% epiboly stage embryo-injected with 10 ng StdMO. The prechordal plate cells were migrating anteriorly to the top.
(AVI)

Movie S4 **Time-lapse imaging of cellular migration and protrusions in involuting prechordal plate cells of a** ***ptenb*** **tMO_1_-injected morphant.** Shown here is a 5-min movie at 5-s intervals DIC time-lapse image sequence of a 90% epiboly stage embryo-injected with 10 ng *ptenb* tMO_1_. The prechordal plate cells were migrating anteriorly to the top.
(AVI)

Movie S5 **Time-lapse confocal imaging of cells treated with StdMO or** ***ptenb*** **tMO_1_ transplanted into an untreated or** ***ptenb*** **tMO_1_-treated embryo.** Shown here is a 10-min movie at 10-s intervals with 4 combined time-lapse confocal image sequences as designated. The transplanted cells were migrating toward the vegetal pole. (A) STD>UT: StdMO treated cells in an untreated host. (B) STD>MO: StdMO treated cells in a tMO_1_ morphant. (C) MO>UT: tMO_1_ treated cells in an untreated host. (D) MO>MO: tMO_1_ treated cells in a tMO_1_ morphant.
(AVI)
