# Supplementary material for: Ptenb Mediates Gastrulation Cell Movements via Cdc42/AKT1 in Zebrafish
Source: PLoS One. 2011 Apr 11;6(4):e18702. doi: 10.1371/journal.pone.0018702 (PMC3073981; doi:10.1371/journal.pone.0018702)
Supplement: Figure S1 — Embryos were injected with 10 ng of StdMO with or without 25 pg of T7 NCdc42 mRNAs and treated as described in Table 1 . The somite width and extension angle of each embryo were measured and shown (A). Embryos were injected with 10 ng of StdMO without (B) or with 50 pg of T7 NRac1 mRNAs (C), incubated to 10-somite stage and photographed. The percentages of normal and abnormal embryos in each treatment are shown (D). (PDF) [file pone.0018702.s001.pdf]

A

	Somite width (μm)	Extension angle (°)
StdMO 10 ng	192.0 ± 4.6[a] (n = 124)	71.2 ± 4.0[a] (n = 124)
StdMO 10 ng + T17NCdc42 25 pg	192.6 ± 4.0[a] (n = 92)	79.3 ± 4.6[b] (n = 92)

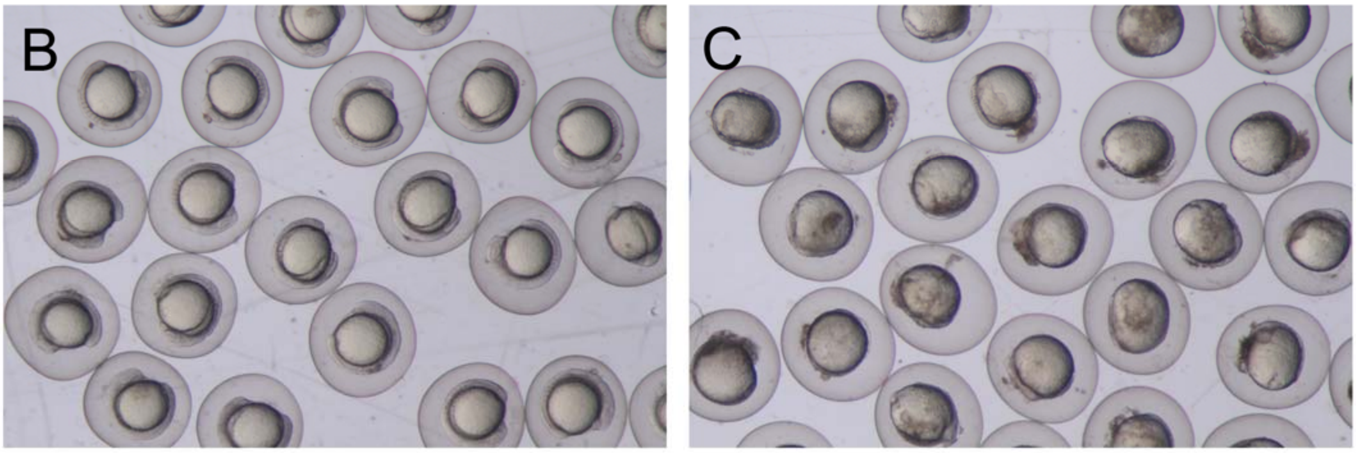

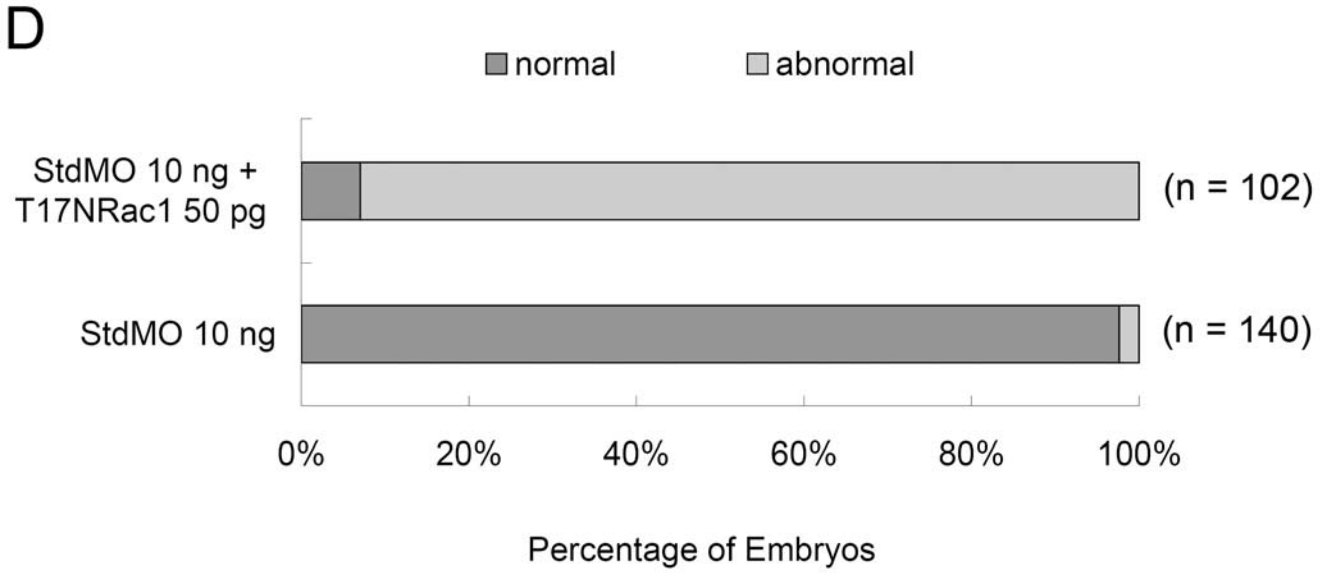

Figure. S1. Embryos were injected with 10 ng of StdMO with or without 25 pg of T7 NCdc42 and treated as described in Table 1. The somite width and extension angle of each embryo were measured and shown (A). Embryos were injected with 10 ng of StdMO without (B) or with 50 pg of T7 NRac1 (C), incubated to 10-somite stage and photographed. The percentages of normal and abnormal embryos in each treatment are shown (D).
